# Inhaled hinoki cypress essential oil improves saliva secretion and swallowing function in older adults with dysphagia: a randomized crossover study

**DOI:** 10.1265/ehpm.25-00319

**Published:** 2026-01-17

**Authors:** Yoichiro Aoyagi, Ryo Furuya, Masaki Kawasaki, Masako Takenouchi, Miho Ohashi, Qing Li

**Affiliations:** 1 Department of Rehabilitation Medicine, Graduate School of Medicine, Nippon Medical School, 1-1-5 Sendagi, Bunkyo-ku, Tokyo 113-8603, Japan; 2 Geriatric Health Services Facility, Misato Care Center, 260-2 Minamihasunuma, Misato-shi, Saitama 341-0028, Japan

**Keywords:** Dysphagia, Olfactory stimulation, Hinoki cypress essential oil, Saliva secretion

## Abstract

**Background:**

Olfactory stimulation with hinoki cypress essential oil reportedly reduces stress hormones and enhances parasympathetic activity, which may in turn increase salivary secretion and facilitate swallowing. However, its effects on swallowing in older adults with dysphagia remain unclear.

**Methods:**

In this placebo-controlled, single-blind, randomized crossover study, older adults with dysphagia (Food Intake Level Scale ≤9) underwent 5-minute olfactory stimulation with hinoki cypress essential oil or rice oil (placebo) in two sessions one week apart. Repetitive saliva swallowing test (RSST), 3 mL modified water swallowing test (MWST), 30 mL water swallowing test (WST), saliva secretion for 5 minutes, blood pressure, and heart rate were measured before and after each stimulation. Changes from pre- to post-stimulation were compared within each condition and between conditions using Wilcoxon’s signed-rank test.

**Results:**

Thirty-four participants (47% women; 80 ± 10 years) were included. Hinoki cypress essential oil significantly improved RSST (median difference 0.5, 95% CI 0.0–1.0; p = 0.004), MWST (0.5, 95% CI 0.0–1.0; p = 0.003), and saliva secretion (1.0 mL, 95% CI 0.5–2.0; p = 0.002), whereas placebo produced no significant change (all p > 0.05). Improvements in swallowing function were greater with hinoki than with placebo. The increase in saliva secretion following hinoki stimulation was not associated with changes in RSST or MWST scores.

**Conclusions:**

Brief olfactory stimulation with hinoki cypress essential oil improved swallowing function and increased saliva secretion in older adults with dysphagia compared with placebo in a randomized crossover design. Hinoki cypress essential oil inhalation may represent a simple, noninvasive adjunctive strategy for dysphagia management, warranting confirmation in larger and longer trials.

**Trial registration:**

This study was registered in the UMIN Clinical Trials Registry (UMIN-CTR) under registration number UMIN000053271.

**Supplementary information:**

The online version contains supplementary material available at https://doi.org/10.1265/ehpm.25-00319.

## Graphical Abstract

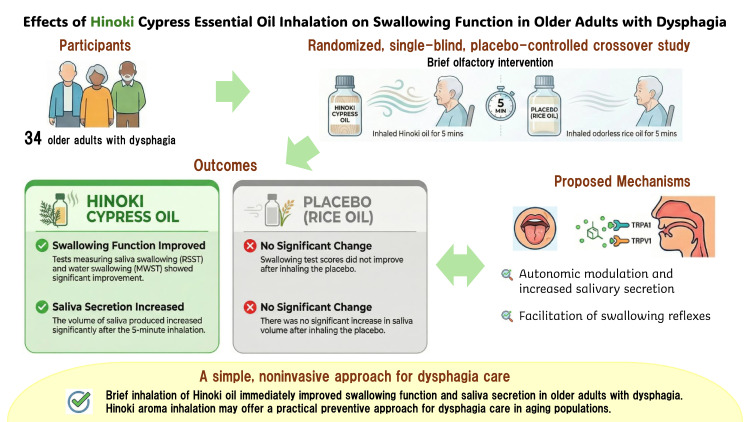

## Introduction

Forest bathing (Shinrin-yoku) has been proposed as a health-promoting exposure to forest environments and is known to reduce stress hormones, increase parasympathetic nerve activity, and decrease sympathetic nerve activity, stabilizing the balance of the autonomic nervous system [1]. People enjoy forest bathing through all five senses [2, 3], including olfaction, by breathing in volatile organic substances called phytoncides such as α-pinene, limonene, and eugenol emitted from trees [4–6]. These phytoncides modulate stress responses and immune function in humans.

Dysphagia in older adults is influenced not only by structural and neuromuscular changes but also by autonomic regulation and sensory input to the swallowing network. Parasympathetic dominance increases salivary secretion [8], which facilitates bolus formation and lubrication, and sensory stimulation of the oropharynx can enhance the swallowing reflex. Hinoki cypress essential oil is rich in phytoncides and has been reported to increase parasympathetic activity [7] and to reduce psychological stress in clinical studies [6, 7]. Thus, olfactory stimulation with hinoki cypress essential oil may improve swallowing function by (1) increasing salivary secretion through parasympathetic activation and (2) enhancing oropharyngeal sensory input.

Plant-derived volatile compounds such as α-pinene and limonene can activate transient receptor potential (TRP) channels, including TRP ankyrin-1 (TRPA1) and TRP vanilloid 1 (TRPV1). TRPA1 and TRPV1 are widely expressed in the human oropharynx [9], suggesting that inhaled phytoncides may modulate sensory pathways relevant to swallowing. In addition, aging and chronic inflammation are associated with olfactory dysfunction [10, 11], which may attenuate responsiveness to olfactory interventions in older adults.

Based on these considerations, we hypothesized that inhalation of hinoki cypress essential oil would acutely increase salivary secretion and improve the swallowing function in older adults with dysphagia compared with an odorless placebo in a randomized crossover design.

## Methods

### Participants

Residents of the Misato Care Center, a geriatric health service facility in Japan, with dysphagia were recruited. Swallowing ability was classified using the Food Intake LEVEL Scale (FILS) [12]. FILS is a 10-point ordinal scale ranging from level 1 (no oral intake) to level 10 (normal oral intake without restrictions), with higher scores indicating better oral intake ability. Participants with FILS ≤ 9 were considered to have dysphagia and were eligible for inclusion in this study. Residents who scored ≤15 on the mini-mental state examination (MMSE) were excluded because they might be unable to provide informed consent or remain still during a 5-minute olfactory stimulation. This study was approved by the ethics committee of the Misato Care Center (approval ID: MC202101) in accordance with the Declaration of Helsinki. Written informed consent was obtained from all participants after a full explanation of the study procedures.

### Clinical characteristics

Data on age, sex, and primary disease that caused admission to the facility were collected from medical records. Primary diseases were classified into six categories: cerebrovascular disease, neurodegenerative disease, musculoskeletal disease, neuromuscular disease, head and neck cancer, and others. A speech-language therapist evaluated the FILS and MMSE scores.

### Study design and olfactory stimulation

**Fig. 1:**
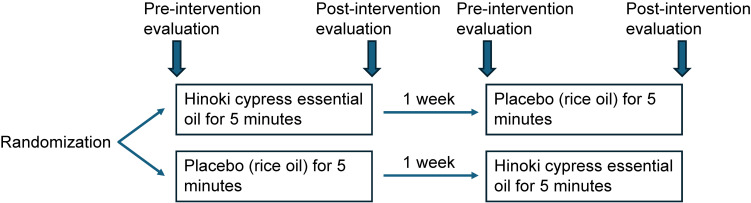
Study design. A randomized crossover study was conducted on older people with decreased swallowing function.

This placebo-controlled, single-blind, crossover study was conducted at a geriatric health service facility (Fig. 1). Absorbent cotton of 7.5 cm^2^ (Hospital gauze^®^, Osaki Medical Co., Japan) was soaked with 150 µL of hinoki cypress essential oil (100% pure oil distilled from *Chamaecyparis obtusa* provided by Hinoki Seiko Co., Japan) or rice oil (Komeabura^®^, Boso Oil and Fat Co., Ltd., Japan). Rice oil was used as the placebo because it has low volatility and minimal characteristic odor compared with hinoki cypress essential oil, making it suitable as an olfactory control in this exploratory study. Each participant was randomized into either the hinoki cypress essential oil or placebo group using a dice.

The participants were seated and relaxed, and their external nostrils were covered with absorbent cotton for olfactory stimulation (Supplementary Fig. 1S). Olfactory stimulation was performed for 5 minutes. To blind the participants, they were informed that they might experience olfactory sensations through the absorbent cotton, regardless of group assignment. Another olfactory stimulation was performed for 5 minutes one week later.

### Outcomes

A speech-language therapist performed the repetitive saliva swallowing test (RSST) [13, 14], modified water swallowing test (MWST) [15, 16], and 30 mL water swallowing test (30 mL WST) [17, 18], and measured the volume of salivary secretion, blood pressure, and heart rate just before and after each olfactory stimulation.

The RSST is a screening test wherein a patient is instructed to swallow saliva as many times as possible in 30 seconds, while deglutition is assessed through palpation of the larynx. Two or fewer dry swallows detected within 30 seconds were considered abnormal. In MWST, 3 mL of cold water is administered to the oral vestibule of a patient, who was then instructed to swallow it. If a patient could not swallow or had dyspnea, coughing, or wet-hoarse dysphonia, an appropriate score was assigned (1 for inability to swallow, 2 for dyspnea, and 3 for cough or dysphonia), and the test was completed. Otherwise, the participants were instructed to perform two dry swallows. If water could be swallowed, neither of the two dry swallows could be performed within 30 seconds, and a score of 4 was given. A score of 5 was assigned to those with the ability to swallow water and two dry swallows. Only when the MWST score was ≧4, 30 mL WST was performed. 30 mL WST results were classified as follows: grade 1: no interruption, no choking or coughing; grade 2: two or more interruptions, no choking or coughing; grade 3: no interruption, with coughing; grade 4: more than two interruptions, with choking; grade 5: frequent cough, cannot swallow entirely. The salivary secretion volume was measured by ejecting the saliva gathered from the mouth into a test tube for 5 minutes [19]. This study was registered in the UMIN Clinical Trials Registry (UMIN-CTR) under registration number UMIN000053271.

### Sample size considerations and statistical analysis

This study was designed as an exploratory randomized crossover trial. Because each participant served as their own control, the crossover design increased statistical efficiency even with a modest sample size. Given the ordinal and non-normally distributed nature of the swallowing outcomes, nonparametric paired tests were used. In addition, effect sizes and 95% confidence intervals were reported to supplement p values and to allow interpretation of the magnitude and precision of the observed effects. The present sample size represents all eligible residents during the study period and was considered adequate for this exploratory investigation.

Wilcoxon’s signed-rank test was used to compare the RSST, MWST, 30 mL WST, saliva secretion volume, blood pressure, and heart rate before and after the two olfactory stimulations. Within each condition (hinoki cypress essential oil or placebo), pre- and post-stimulation values were compared using Wilcoxon’s signed-rank test. To account for the crossover design, change scores (post-pre) were calculated for each outcome under both conditions, and the changes with hinoki cypress essential oil were compared with those with placebo using Wilcoxon’s signed-rank test for paired data. Effect sizes (e.g., median differences in change between conditions) and corresponding 95% confidence intervals were calculated for the main outcomes. Median differences and 95% confidence intervals were estimated by the Hodges–Lehmann method.

Spearman’s rank correlation coefficient was also assessed to establish the association between changes in saliva secretion volume, RSST score, and MWST score before and after olfactory stimulation with hinoki cypress essential oil. All statistical analyses were performed using SPSS version 29 (IBM Corp., Armonk, NY, USA). Statistical significance was set at p < 0.05, and data were expressed as median (interquartile range) except for age and MMSE.

## Results

Thirty-four subjects (16 women, 80.2 ± 10.2 years old) participated in this study. The median FILS of the participants was 8 (6.75–8), and the average ± standard deviation of the MMSE was 23.9 ± 5.2 (Table 1). The primary diseases that led to admission to the facility were cerebrovascular disease (44%), neurodegenerative disease (18%), musculoskeletal disease (18%), neuromuscular disease (6%), head and neck cancer (3%), and others (44%).

**Table 1:** Demographic characteristics of 34 participants.

Age (years)	80.2 ± 10.2
Gender (Male, %)	18, 53%
MMSE	23.9 ± 5.2
FILS	8 (6.75–8.00)
Underlying disease (n, %)	
Cerebrovascular disease	15, 44%
Neurodegenerative disease	6, 18%
Musculoskeletal disease	6, 18%
Neuromuscular disease	2, 6%
Head and neck cancer	1, 3%
Others	4, 12%

Data are presented as n, %, mean ± standard deviation, or median (interquartile range).

Abbreviation: MMSE, mini-mental state examination; FILS, functional intake level scale

At the pre-intervention baseline evaluation, no statistical difference was observed in RSST, MWST, 30 mL WST, saliva secretion volume, blood pressure, or heart rate between the hinoki cypress essential oil and placebo groups. Table 2 summarizes the pre- and post-intervention values and the within-condition and between-condition differences.

**Table 2:** Effects of olfactory stimulation with hinoki cypress essential oil compared with placebo.

	**Hinoki (cypress essential oil)**				**Placebo (rice oil)**				**ΔHinoki − ΔPlacebo**	

	**Pre-intervention** **(interquartile range)**	**Post-intervention** **(interquartile range)**	**Median Difference** **[95% CI]**	***p* value**	**Pre-intervention** **(interquartile range)**	**Post-intervention** **(interquartile range)**	**Median Difference** **[95% CI]**	***p* value**	**Median Difference** **[95% CI]**	***p* value**
RSST (number of times)	2 (1–3)	2.5 (1–3)	0.5 [0.0 to 1.0]	0.004	2 (1–3)	2 (1–3)	0.0 [−0.5 to 0.0]	0.067	1.0 [0.5 to 1.5]	0.003
3 mL MWST (score)	4 (3–4)	4 (4–5)	0.5 [0.0 to 1.0]	0.003	4 (3–4)	4 (3–4)	0.0 [0.0 to 0.0]	0.739	0.5 [0.0 to 1.0]	0.009
30 mL WST (grade)	2 (2–3)	2 (2–3)	0.0 [−0.5 to 1.0]	0.167	2 (2–3.5)	2 (2–3.5)	0.0 [0.0 to 0.0]	0.257	0.0 [−0.5 to 0.0]	0.408
Saliva secretion (ml)	2 (0–6)	4 (1–8)	1.0 [0.5 to 2.0]	0.002	2 (0.8–6.5)	2.5 (1–6)	0.0 [0.0 to 1.0]	0.231	0.6 [0.0 to 1.5]	0.056
SBP (mmHg)	123 (111–140)	127 (117–140)	1.0 [−2.0 to 6.5]	0.488	125 (109–136)	129.5 (112–140.25)	4.5 [−0.5 to 8.0]	0.067	−1.0 [−8.0 to 4.5]	0.567
DBP (mmHg)	70.5 (62.75–79)	72 (65.5–79.5)	2.5 [0.0 to 5.0]	0.046	70 (62–77.25)	72.5 (66–81)	2.5 [0.5 to 5.0]	0.025	0.0 [−4.0 to 3.5]	1.000
HR (bpm)	73 (65.75–78.5)	70 (65.5–81)	−0.5 [−2.0 to 0.5]	0.319	70 (65.75–77.5)	69.5 (63.5–82.25)	0.0 [−1.5 to 2.5]	0.822	−1.0 [−3.0 to 1.5]	0.348

• Pre- and post-intervention values, within-condition changes, and between-condition differences for swallowing function, salivary secretion, and physiological parameters are presented as median (interquartile range).

• Median Difference represents the Hodges–Lehmann estimator of the median paired difference with 95% confidence interval.

• p values for within-condition comparisons (pre vs. post) and between-condition differences (ΔHinoki − ΔPlacebo) were obtained using the Wilcoxon signed-rank test.

• ΔHinoki − ΔPlacebo represents the difference in pre-to-post changes between the hinoki and placebo sessions in the crossover design.

Abbreviations: RSST, repetitive saliva swallowing test; MWST, modified water swallowing test; WST, water swallowing test; SBP, systolic blood pressure; DBP, diastolic blood pressure; HR, heart rate

### Swallowing function

**Fig. 2:**
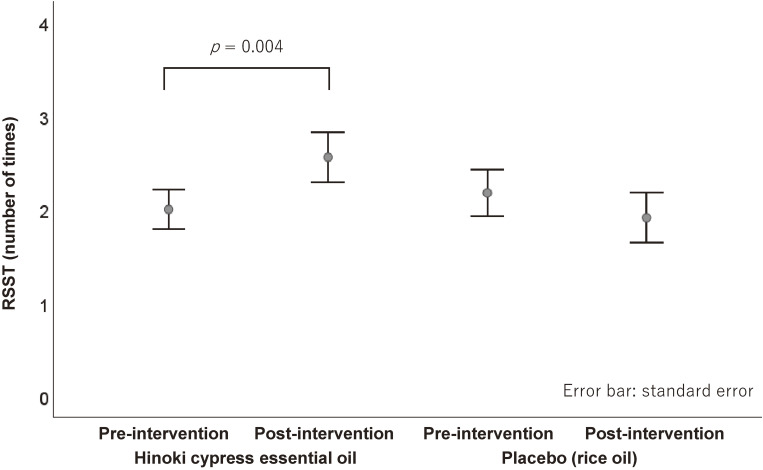
Comparison of repetitive saliva swallowing test (RSST) scores at pre- and post-intervention.

**Fig. 3:**
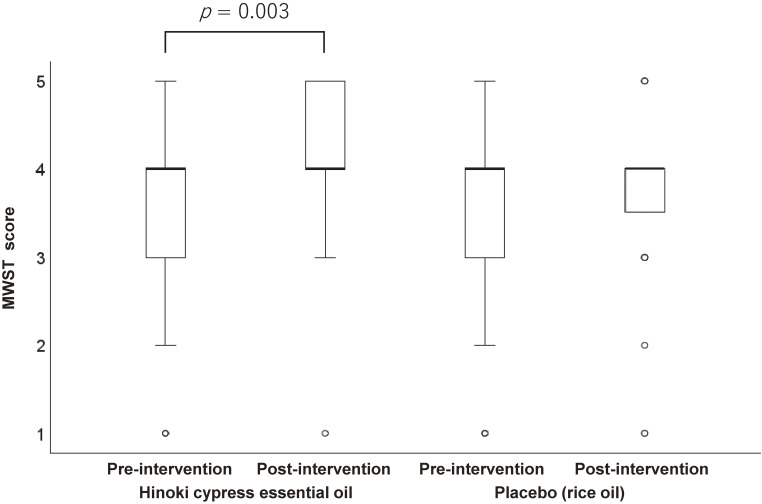
Boxplot of 3 mL modified water swallowing test (MWST) scores at pre- and post-intervention.

All the participants completed the study protocol without adverse events. Olfactory stimulation with hinoki cypress essential oil significantly improved swallowing performance. RSST increased from a median of 2 (IQR 1–3) to 2.5 (1–3) swallows (median difference 0.5, 95% CI 0.0–1.0; p = 0.004) (Fig. 2). Similarly, MWST improved from 4 (3–4) to 4 (3–5) points (median difference 0.5, 95% CI 0.0–1.0; p = 0.003) (Fig. 3). In contrast, placebo stimulation produced no significant change in RSST or MWST.

Direct comparison of pre-to-post changes showed significantly greater improvements with hinoki than with placebo for both RSST (median difference in Δ = 1.0, 95% CI 0.5–1.5; p = 0.003) and MWST (median difference in Δ = 0.5, 95% CI 0.0–1.0; p = 0.009). For the 30 mL WST, neither session produced a significant pre–post change, and the between-condition difference was not significant.

### Saliva secretion

**Fig. 4:**
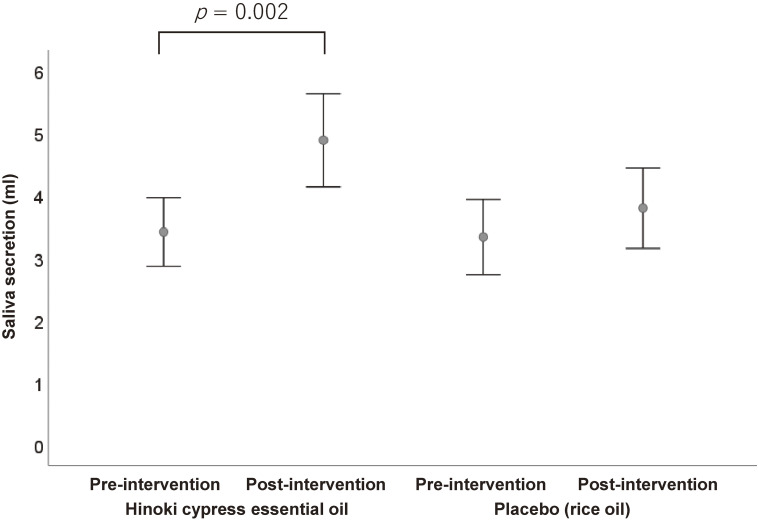
Comparison of saliva secretion volume at pre- and post-intervention. Error bars show standard error of the median.

Hinoki stimulation significantly increased saliva secretion volume from 2 (0–6) mL to 4 (1–8) mL (median difference 1.0, 95% CI 0.5–2.0; p = 0.002), whereas placebo produced no significant change (Fig. 4).

### Other parameters

Additionally, to clarify the association between changes in saliva secretion volume and swallowing function, we further analyzed them using Spearman’s rank correlation coefficient. The individual change in saliva secretion volume after olfactory stimulation with hinoki cypress essential oil was not associated with the change in RSST scores (p = 0.268, r = 0.195) (Supplementary Fig. 2S). Similarly, individual changes in salivary secretion volume were not associated with changes in MWST scores (p = 0.963, r = 0.008) (Supplementary Fig. 3S).

Both olfactory stimulation with hinoki cypress essential oil and rice oil inhalation slightly increased diastolic blood pressure but did not affect systolic blood pressure or heart rate.

## Discussion

Olfactory stimulation with hinoki cypress essential oil for 5 min immediately improved the RSST, MWST, and saliva secretion volume scores, whereas olfactory stimulation with a placebo did not improve any swallow-related parameters in a randomized crossover study in individuals with decreased swallowing function. It was also found that the increase in saliva secretion volume after olfactory stimulation with hinoki cypress essential oil was not significantly associated with the improved RSST or MWST scores representing swallowing function. This means that, beyond hypothetical speculation, the improved swallowing function was not simply due to increased salivary secretion. Plausible biochemical and physiological mechanisms for improving swallowing function and saliva secretion are discussed below.

### Swallowing function

Hinoki cypress essential oil includes volatile organic substances, called phytoncides from trees, such as α-pinene, limonene, and eugenol [4–6]. A plant-derived biogenic volatile organic compound, α-pinene, activates transient receptor potential ankyrin-1 (TRPA1) in mice [20]. Limonene, a monoterpene found in the essential oils of various plants, is known for its antitumor and anti-inflammatory properties and activates TRPA1 in mice [21]. Eugenol activated transient receptor potential vanilloid 1 (TRPV1) [22]. TRPV1 and TRPA1 are widely expressed in the oropharynx. Inhaling phytoncides such as α-pinene, limonene, and eugenol may improve swallowing reflexes through TRPA1 and TRPV1 in humans as in animal models. Previous studies have found that capsaicinoids (TRPV1 agonists) [23] and piperine (TRPA1 and TRPV1 agonists) [24] improve the swallowing response in patients with oropharyngeal dysphagia. Oropharyngeal TRPA1 and TRPV1 receptors appear to be promising therapeutic targets for the development of active treatments for patients with oropharyngeal dysphagia.

### Saliva secretion

Saliva secretion increases when the parasympathetic nervous system becomes dominant and salivary glands are stimulated [25]. It has been reported that touching hinoki wood or smelling hinoki cypress essential oil increases the high-frequency component of heart rate variability, indicating an increase in parasympathetic nervous activity [26, 27]. It has also been reported that the phytoncide fragrance of hinoki cypress essential oil improves parasympathetic nerve activity in cancer survivors [7]. Similarly, the most plausible explanation that olfactory stimulation with hinoki cypress essential oil leads to increased saliva production is that inhalable phytoncides increase the parasympathetic activity [7]. Saliva protects the teeth and oropharyngeal mucosa, facilitates speech articulation, and is imperative for mastication and swallowing [25, 28].

Although heart rate did not change, it may not be sensitive enough to detect subtle parasympathetic shifts induced by brief olfactory stimulation. More sensitive measures, such as heart rate variability (HF component) or pupillometry, may be required to capture autonomic modulation in future studies.

### Dissociation in swallowing function and saliva secretion

Interestingly, although hinoki cypress essential oil significantly increased salivary secretion, individual changes in salivary flow did not correlate with changes in RSST or MWST scores. This suggests that the beneficial effect on swallowing is not mediated solely by increased salivary volume. Swallowing is controlled by a central pattern generator that integrates multisensory input from the oropharynx and is modulated by autonomic and cortical influences. It is therefore plausible that olfactory stimulation with hinoki cypress essential oil enhances the excitability of the swallowing network through sensory and autonomic pathways, while only modestly affecting salivary volume beyond a threshold that is sufficient for lubrication.

### Limitations of this study and future perspectives

This study had several limitations. First, the sample size was modest and recruited from a single geriatric health facility, and the results should be considered exploratory. Second, we did not measure olfactory thresholds, chronic inflammatory markers, or detailed indices of autonomic function such as heart rate variability. Given the known impacts of aging and chronic inflammation on olfaction [29], and the likely involvement of autonomic and inflammatory pathways in the response to hinoki cypress essential oil, future studies should include these mediators to elucidate the underlying mechanisms. Third, although rice oil was chosen as a neutral placebo because of its minimal odor, we did not formally assess participants’ ability to distinguish hinoki from rice oil; blinding adequacy should be evaluated in future trials. Fourth, this study did not assess potential mediators such as inflammatory activity, autonomic regulation, or TRP channel activation, which may underlie the observed effects. Future mechanistic studies are warranted to clarify these pathways. Furthermore, we assessed only the immediate effects after a single 5-minute exposure and focused on saliva swallowing and thin-liquid tests; longer-term interventions and evaluations with semi-solid and solid boluses should be conducted to determine the durability and generalizability of the observed improvements. Finally, comparative trials using established sensory/olfactory stimulants, such as capsaicin or black pepper oil, would be valuable for positioning hinoki cypress essential oil relative to existing interventions and for identifying patient subgroups who may benefit most.

In conclusion, the immediate improvement observed after olfactory stimulation with hinoki cypress essential oil indicates its potential efficacy in enhancing saliva secretion and swallowing function, possibly due to parasympathetic stimulation and activation of TRPA1/V1 receptors. This finding opens up possibilities for further research, such as multicenter studies and investigations of long-term improvement effects. If confirmed, olfactory stimulation may represent a practical adjunct for dysphagia care in settings where more invasive interventions are challenging to implement.

## Supplementary information

**Additional file 1:** **Supplementary Figure 1S.** Participant covered their external nostrils with absorbent cotton with 7.5 cm square for olfactory stimulation. **Supplementary Figure 2S.** Scatter plots of changes in saliva secretion volume and RSST before and after the olfactory stimulation. Abbreviation: RSST, repetitive saliva swallowing test. **Supplementary Figure 3S.** Scatter plots of changes in saliva secretion volume and MWST score before and after the olfactory stimulation. Abbreviation: MWST, modified water swallowing test.

## Declarations

### Availability of data and material

The datasets generated and/or analyzed during the current study are available from the corresponding author upon reasonable request.

## References

[1] Li Q. Effects of forest environment (Shinrin-yoku/Forest bathing) on health promotion and disease prevention -the Establishment of “Forest Medicine”. Environ Health Prev Med. 2022;27:43. doi: 10.1265/ehpm.22-00160. 36328581 PMC9665958

[2] Li Q. Forest bathing: how trees can help you find health and happiness. New York, New York: Viking; 2018.

[3] Li Q, Morimoto K, Nakadai A, Inagaki H, Katsumata M, Shimizu T, . Forest bathing enhances human natural killer activity and expression of anti-cancer proteins. Int J Immunopathol Pharmacol. 2007;20:3–8. doi: 10.1177/03946320070200S202. 17903349

[4] Lee SJ, Han JI, Lee GS, Park MJ, Choi IG, Na KJ, . Antifungal effect of eugenol and nerolidol against Microsporum gypseum in a guinea pig model. Biol Pharm Bull. 2007;30:184–8. doi: 10.1248/bpb.30.184. 17202684

[5] Li Q, Nakadai A, Matsushima H, Miyazaki Y, Krensky AM, Kawada T, . Phytoncides (wood essential oils) induce human natural killer cell activity. Immunopharmacol Immunotoxicol. 2006;28:319–33. doi: 10.1080/08923970600809439. 16873099

[6] Li Q, Kobayashi M, Wakayama Y, Inagaki H, Katsumata M, Hirata Y, . Effect of phytoncide from trees on human natural killer cell function. Int J Immunopathol Pharmacol. 2009;22:951–9. doi: 10.1177/039463200902200410. 20074458

[7] Heo SJ, Park SK, Jee YS. Effects of phytoncide on immune cells and psychological stress of gynecological cancer survivors: randomized controlled trials. J Exerc Rehabil. 2023;19:170–80. doi: 10.12965/jer.2346150.075. 37435591 PMC10331144

[8] Endoh T. Modulation of voltage-dependent calcium channels by neurotransmitters and neuropeptides in parasympathetic submandibular ganglion neurons. Arch Oral Biol. 2004;49:539–57. doi: 10.1016/j.archoralbio.2004.02.005. 15126136

[9] Alvarez-Berdugo D, Rofes L, Farre R, Casamitjana JF, Enrique A, Chamizo J, . Localization and expression of TRPV1 and TRPA1 in the human oropharynx and larynx. Neurogastroenterol Motil. 2016;28:91–100. doi: 10.1111/nmo.12701. 26530852

[10] Xie Y, Wang S, Cha X, Li F, Xu Z, Wu J, . Aging and chronic inflammation: impacts on olfactory dysfunction-a comprehensive review. Cell Mol Life Sci. 2025;82:199. doi: 10.1007/s00018-025-05637-5. 40355677 PMC12069206

[11] Kovacs T. Mechanisms of olfactory dysfunction in aging and neurodegenerative disorders. Ageing Res Rev. 2004;3:215–32. doi: 10.1016/j.arr.2003.10.003. 15177056

[12] Kunieda K, Ohno T, Fujishima I, Hojo K, Morita T. Reliability and validity of a tool to measure the severity of dysphagia: the Food Intake LEVEL Scale. J Pain Symptom Manage. 2013;46:201–6. doi: 10.1016/j.jpainsymman.2012.07.020. 23159683

[13] Oguchi K, Saitoh E, Baba M, Kusudo M, Tanaka T, Onogi K. The repetitive saliva swallowing test (RSST) as a screening test of functional dysphagia (2) Validity of RSST. Jpn J Rehabil Med. 2000;37:383–8 (In Japanese. Abstract in English).

[14] Persson E, Wardh I, Ostberg P. Repetitive Saliva Swallowing Test: Norms, Clinical Relevance and the Impact of Saliva Secretion. Dysphagia. 2019;34:271–8. doi: 10.1007/s00455-018-9937-0. 30132122 PMC6421277

[15] Osawa A, Maeshima S, Tanahashi N. Water-swallowing test: screening for aspiration in stroke patients. Cerebrovasc Dis. 2013;35:276–81. doi: 10.1159/000348683. 23548854

[16] Tohara H, Saitoh E, Mays KA, Kuhlemeier K, Palmer JB. Three tests for predicting aspiration without videofluorography. Dysphagia. 2003;18:126–34. doi: 10.1007/s00455-002-0095-y. 12825906

[17] Horiguchi S, Suzuki Y. Screening tests in evaluating swallowing function. Japan Med Assoc J. 2011;54:31–4.

[18] Hou P, Deng H, Wu Z, Liu H, Liu N, Zheng Z, . Detection of salivary aspiration using radionuclide salivagram SPECT/CT in patients with COPD exacerbation: a preliminary study. J Thorac Dis. 2016;8:2730–7. doi: 10.21037/jtd.2016.09.63. 27867548 PMC5107550

[19] Mizuhashi F, Koide K. Salivary secretion and salivary stress hormone level changes induced by tongue rotation exercise. J Adv Prosthodont. 2020;12:204–9. doi: 10.4047/jap.2020.12.4.204. 32879710 PMC7449817

[20] Jin L, Xie Z, Lorkiewicz P, Srivastava S, Bhatnagar A, Conklin DJ. Endothelial-dependent relaxation of alpha-pinene and two metabolites, myrtenol and verbenol, in isolated murine blood vessels. Am J Physiol Heart Circ Physiol. 2023;325:H1446–60. doi: 10.1152/ajpheart.00380.2023. 37889254 PMC11584230

[21] Kaimoto T, Hatakeyama Y, Takahashi K, Imagawa T, Tominaga M, Ohta T. Involvement of transient receptor potential A1 channel in algesic and analgesic actions of the organic compound limonene. Eur J Pain. 2016;20:1155–65. doi: 10.1002/ejp.840. 27030509

[22] Ye H, Lin Q, Mei Q, Liu Q, Cao S. Study on mechanism of transdermal administration of eugenol for pain treatment by network pharmacology and molecular docking technology. Heliyon. 2024;10:e29722. doi: 10.1016/j.heliyon.2024.e29722. 38681628 PMC11046106

[23] Rofes L, Arreola V, Martin A, Clave P. Natural capsaicinoids improve swallow response in older patients with oropharyngeal dysphagia. Gut. 2013;62:1280–7. doi: 10.1136/gutjnl-2011-300753. 22722616

[24] Rofes L, Arreola V, Martin A, Clave P. Effect of oral piperine on the swallow response of patients with oropharyngeal dysphagia. J Gastroenterol. 2014;49:1517–23. doi: 10.1007/s00535-013-0920-0. 24326980

[25] Pedersen AML, Sorensen CE, Proctor GB, Carpenter GH, Ekstrom J. Salivary secretion in health and disease. J Oral Rehabil. 2018;45:730–46. doi: 10.1111/joor.12664. 29878444

[26] Ikei H, Song C, Miyazaki Y. Physiological effect of olfactory stimulation by Hinoki cypress (Chamaecyparis obtusa) leaf oil. J Physiol Anthropol. 2015;34:44. doi: 10.1186/s40101-015-0082-2. 26694076 PMC4687359

[27] Ikei H, Song C, Miyazaki Y. Physiological Effects of Touching the Wood of Hinoki Cypress (Chamaecyparis obtusa) with the Soles of the Feet. Int J Environ Res Public Health. 2018;15. doi: 10.3390/ijerph15102135. PMC6210085 30274160

[28] Rogus-Pulia NM, Larson C, Mittal BB, Pierce M, Zecker S, Kennelty K, . Effects of Change in Tongue Pressure and Salivary Flow Rate on Swallow Efficiency Following Chemoradiation Treatment for Head and Neck Cancer. Dysphagia. 2016;31:687–96. doi: 10.1007/s00455-016-9733-7. 27492408 PMC5018456

[29] Park Y, Yoo SA, Kim WU, Cho CS, Woo JM, Yoon CH. Anti-inflammatory effects of essential oils extracted from Chamaecyparis obtusa on murine models of inflammation and RAW 264.7 cells. Mol Med Rep. 2016;13:3335–41. doi: 10.3892/mmr.2016.4905. 26936418

