# Supplementary material for: Inhaled hinoki cypress essential oil improves saliva secretion and swallowing function in older adults with dysphagia: a randomized crossover study
Source: Environ Health Prev Med. 2026 Jan 17;31:4. doi: 10.1265/ehpm.25-00319 (PMC12834643; doi:10.1265/ehpm.25-00319)
Supplement: Supplementary file 1 — Additional file 1: Supplementary Figure 1S. Participant covered their external nostrils with absorbent cotton with 7.5 cm square for olfactory stimulation. Supplementary Figure 2S. Scatter plots of changes in saliva secretion volume and RSST before and after the olfactory stimulation. Abbreviation: RSST, repetitive saliva swallowing test. Supplementary Figure 3S. Scatter plots of changes in saliva secretion volume and MWST score before and after the olfactory stimulation. Abbreviation: MWST, modified water swallowing test. [file ehpm-31-004-s001.pptx]

## Slide 1
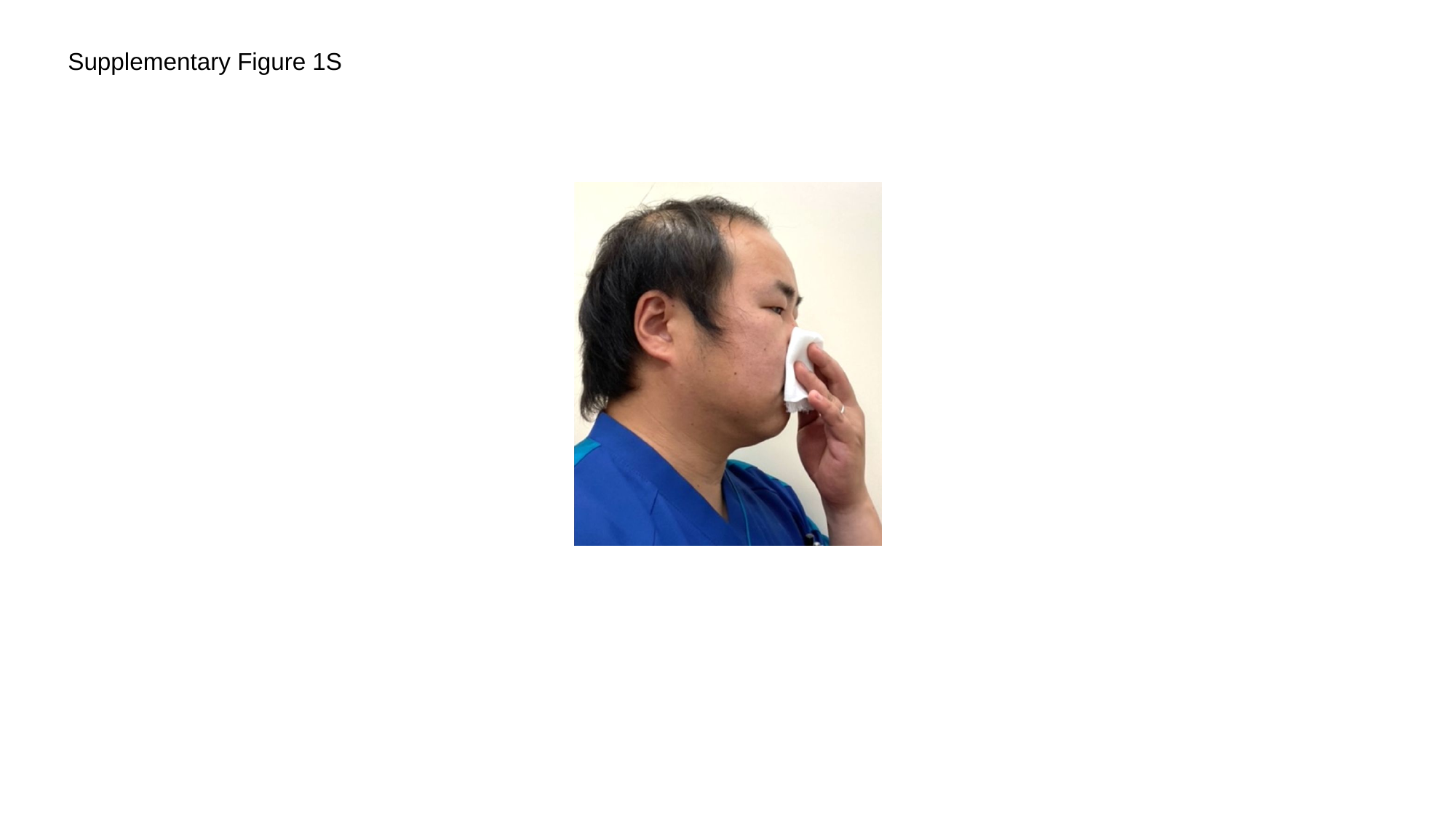

Supplementary Figure 1S

## Slide 2
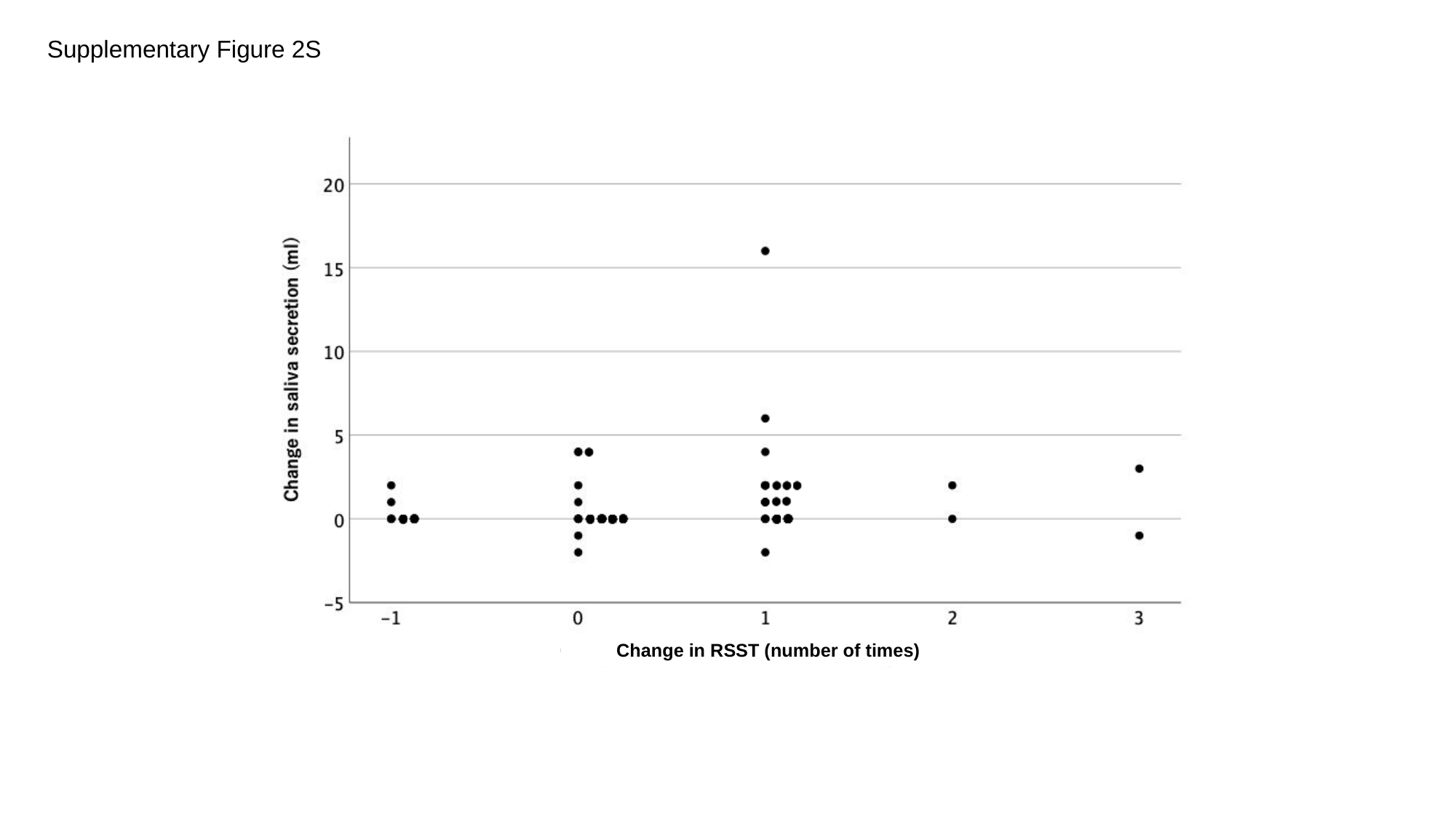

Supplementary Figure 2S
Change in RSST (number of times)

## Slide 3
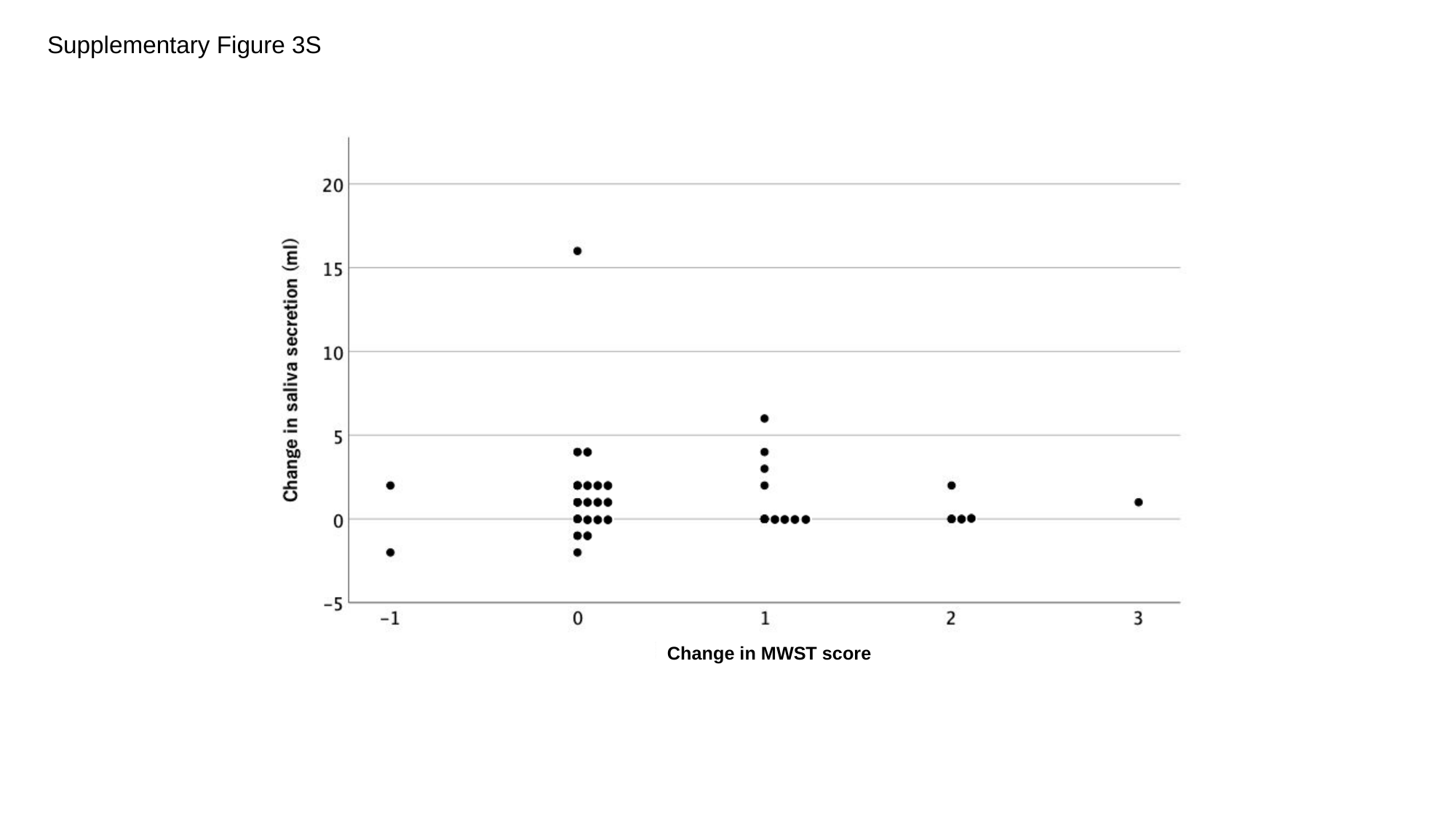

Supplementary Figure 3S
Change in MWST score
